# Breaking down barriers: is intestinal mucus degradation by *Akkermansia muciniphila* beneficial or harmful?

**DOI:** 10.1128/iai.00503-24

**Published:** 2025-08-11

**Authors:** Anna M. Tingler, Melinda A. Engevik

**Affiliations:** 1 Department of Regenerative Medicine & Cell Biology, Medical University of South Carolina 169830, Charleston, South Carolina, USA; 2 Department of Pharmacology & Immunology, Medical University of South Carolina 2345, Charleston, South Carolina, USA; University of California at Santa Cruz, Santa Cruz, California, USA

**Keywords:** mucus, mucin, bacteria, glycosyl hydrolases, glycan

## Abstract

*Akkermansia muciniphila* is a specialized mucin-degrading bacterium that plays a pivotal role in gut health and disease. This review examines the dualistic nature of *A. muciniphila* mucin degradation, exploring its potential benefits and risks. As a mucin specialist, *A. muciniphila* uses glycosyl hydrolases and mucinases to degrade mucins, producing metabolites like short-chain fatty acids (SCFAs), branched-chain fatty acids (BCFAs), succinate, and other compounds. These metabolites benefit host health and cross-feed other commensal microbes, such as butyrate producers. *A. muciniphila* levels are inversely correlated with several disease states, such as obesity, diabetes, and inflammatory states, and administration of *A. muciniphila* has been found by several groups to restore and maintain gut homeostasis. However, under certain conditions, such as low dietary fiber or conditions with an altered gut microbiota, excessive mucin degradation by *A. muciniphila* can compromise the mucus barrier, increasing susceptibility to inflammation, infection, and pathogenic overgrowth. Elevated *A. muciniphila* levels have been associated with various diseases and medications, including graft versus host disease (GVHD) and irradiation, and shown to exacerbate infections by enteric pathogens. The context-dependent effects of *A. muciniphila* and mucin degradation underscore the need for a nuanced understanding of its interactions with the host and microbial community. This review aims to provide a balanced perspective on the implications of gut microbial mucus degradation, highlighting that it can be good, and it can be bad depending on the context.

## MUCUS PRODUCTION AND DEGRADATION

The intestinal epithelium is covered by a protective mucus layer that acts as a semipermeable sieve to limit the interaction of luminal antigens with the host. As reviewed in the cited articles, intestinal mucus is composed of proteins, lipids, electrolytes, and water (1, 2). The viscoelastic properties of mucus are due largely to the presence of large, glycosylated proteins known as mucins. The major gel-forming mucin in the mammalian intestine is MUC2, and in humans, there is an additional gel-forming mucin, MUC5B. Both of these mucins are produced by specialized intestinal cells known as goblet cells (3–6). In goblet cells, MUC2 proteins form homodimers in the endoplasmic reticulum (ER) and are then shuttled to the Golgi apparatus, where they form trimers and are glycosylated. O-linked glycosylation at serine and threonine residues on the mucin protein creates a functionally mature mucus that holds water and maintains hydration (7–11). O-linked glycans have core structures of α- and β-linked N-acetyl-glucosamine, N-acetyl-galactosamine, and galactose. These core structures are elongated and modified by α-linked fucose and sialic acid residues, as reviewed in (12), which create the classic bottle-brush structure of mucus. Glycosylated MUC2 is then packaged into mucus granules, where MUC2 dimers form larger polymers (13). These granules are secreted constitutively and can also be released upon stimulation (5, 6). Secreted mucus undergoes ~1,000-fold volume expansion when bicarbonate rapidly increases the local pH and precipitates Ca^2+^-ions from the mucin (14, 15). In addition to mucins, mucus also contains crosslinking Fc fragment of IgG binding proteins, wound healing-associated trefoil factor 3, metalloenzyme CLCA1, lectin-like protein ZG16, and antimicrobials like RELMβ, y6/Plaur domain-containing protein 8, secretory immunoglobulin A, and antimicrobial proteins, as reviewed in reference 16.

In the small intestine, bicarbonate is largely secreted from the cystic fibrosis transmembrane regulator protein on neighboring enterocytes. In the colon, the goblet cells supply bicarbonate via bestrophin-2 bicarbonate transporters (17). The property of mucus varies by location. In the small intestine, the mucus is unattached and easy to remove (5). In contrast, the colon has a two-layered mucus system (5). As mucus is secreted by colonic goblet cells, it emerges as densely packed mucin polymers that form a structured barrier impermeable to bacteria, referred to as the inner mucus layer. When this mucus migrates away from the epithelial surface, it undergoes proteolytic and glycosidic degradation, leading to expansion of the mucin network and the formation of a looser, more permeable outer mucus layer (5). It has been speculated that this expansion of mucus is driven by the proteolytic cleavage of the cysteine-rich parts of the mucin protein at the C terminus and the capacity of the mucin glycans to bind water, as reviewed in reference 18. Under normal conditions, the outer mucus layer is colonized by gut microbes, while the inner mucus layer remains free of microbes. The thickness of the inner mucus layer is commonly used as an indicator of an intact and functional mucus barrier. Given the relevance of the mucus layer in maintaining human health, there has been renewed interest in understanding the factors that contribute to its maintenance and degradation.

**Fig 1:**
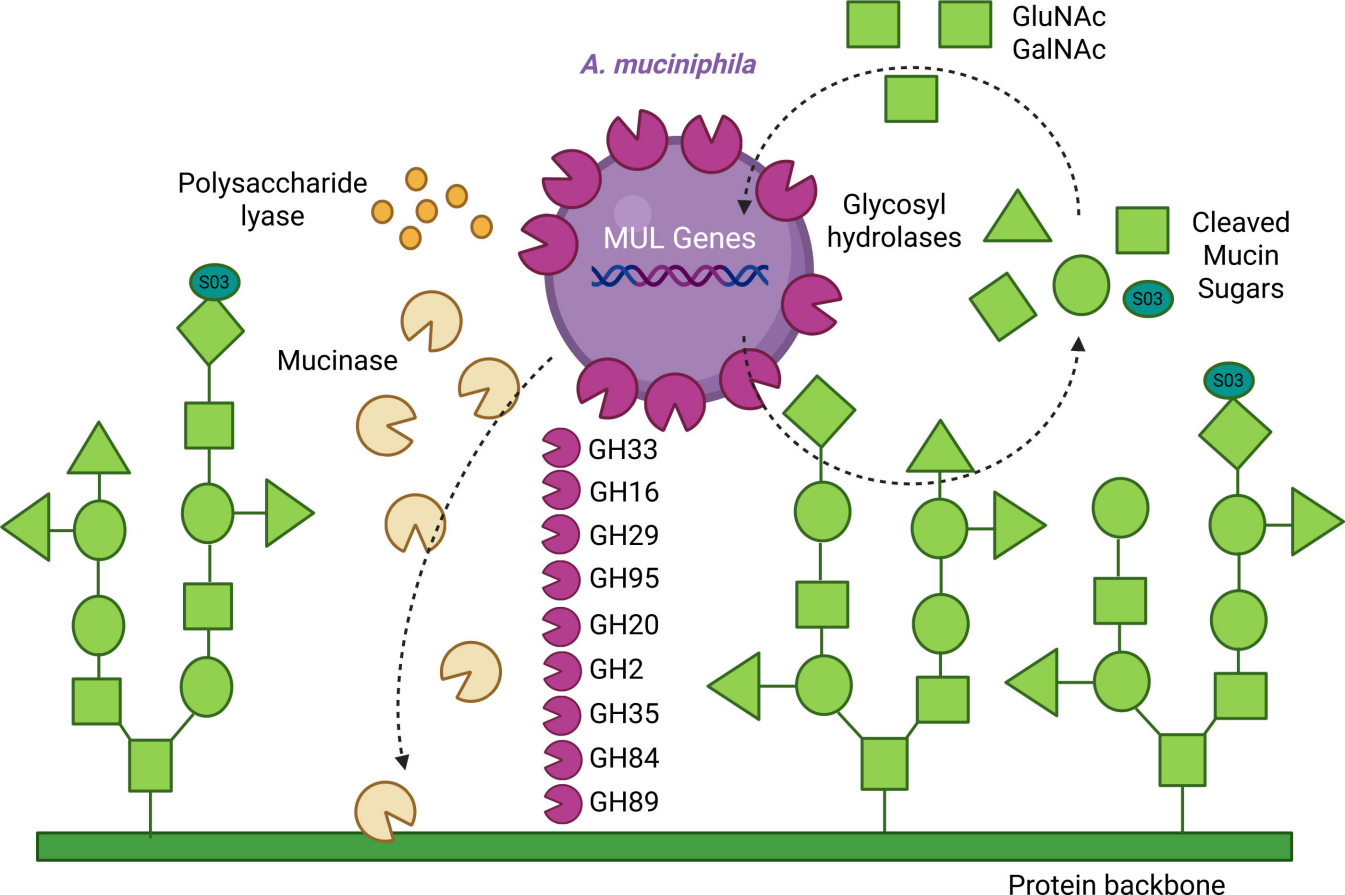
Diagram of *A. muciniphila’s* mucin-degrading capabilities through the use of glycosyl hydrolases (GH33, GH16, GH29, GH95, GH20, GH2, GH35, GH84, and GH89), mucinases, polysaccharide lyases, and mucin utilization locus (MUL) genes.

In addition to functioning as a barrier, lubricant, immune cell signal, and reservoir of signaling peptides, intestinal mucus also acts as a habitat and fuel source for indigenous enteric bacteria (19, 20). Bacteria that encode specific glycosyl hydrolases (GHs) are capable of enzymatically degrading mucin glycans, and the released oligosaccharides can then be used as a nutrient for the mucus-associated microbiota (21, 22). In this way, mucus can provide a sustainable and consistent nutrient supply for not only mucin-degrading microbes but also neighboring non-mucin-degrading microbes and likely dictates the composition of the mucus-associated microbiota (Fig. 1). The degradation of mucin glycans requires the cooperative action of several glycosyl hydrolases (12, 21, 23–25). Mucus degradation starts with the removal of terminal sulfate groups by sulfatases and sialic acid residues by sialidases (also known as neuraminidases), which are found in the GH33 family. Fucose residues can be removed by fucosidases found in the GH29 or GH95 families. Following the removal of these terminal sugars, the inner glycan oligosaccharides can be removed by N-acetyl-glucosaminidases (GH84, GH85, G89, GH20), N-acetyl-galactosaminidases (GH101, GH129), or galactosidases (GH2, GH35, GH42, GH98). Certain bacteria also possess endo-acting O-glycanases (GH16), which can cleave larger glycan structures. It has been speculated that the core GH-ome for mucin degradation includes GH33, 29/95, and 20, and 35, with more extensive degradation of internal glycans requiring 84/85/89 and 101/129. A recent analysis of the CAZyme mucin-degrading profiles of the human gut microbiota revealed multiple mucin-degrading microbes in each major bacterial phylum (25). In addition to glycosyl hydrolases, some bacteria have enzymes that can degrade the mucin protein backbone, or mucinases, which further digest mucus. Bacteria with the largest mucin glycan degrading repertoire included *Akkermansia muciniphila, Akkermansia glycaniphila, Bacteroides thetaiotaomicon, Bifidobacterium bifidum, Bifidobacterium breve, Parabacteroides distasonis, Victivallales* species, and *Ruminococcus* species (25). Intestinal mucus degradation by these gut bacteria is a double-edged sword—it can be both beneficial and harmful, depending on the context and balance within the gut environment. On one hand, controlled mucus degradation enables commensal bacteria to establish a niche and outcompete pathogens (26, 27), contributing to colonization resistance. On the other hand, excessive degradation can compromise the mucus barrier, allowing bacteria to directly contact the intestinal epithelium and trigger inflammatory responses (28, 29). Mucin degradation has been shown to influence disease outcomes related to inflammatory bowel disease, enteric infections, colorectal cancer, metabolic diseases, and more (29–31), highlighting its importance in human health.

## *AKKERMANSIA MUCINIPHILA* AND MUCIN DEGRADATION

The best-characterized mucin-degrading microbe to date is *A. muciniphila. A. muciniphila* was first discovered in 2004 by culturing feces from healthy human donors in anaerobic medium containing gastric mucin as the sole carbon and nitrogen source (32). Using this method, they isolated and identified the “mucin-loving” *A. muciniphila* bacterium. *A. muciniphila* is a Gram-negative, aerotolerant anaerobic, non-motile, non-spore-forming, oval-shaped bacterium from the phylum Verrucomicrobia (33–36). *A. muciniphila* makes up ~1%–3% of the human fecal microbiota and is widely distributed among human populations and the animal kingdom (37–40). *A. muciniphila* can be classified into several clades (AmI–AmIV), and these clades differ in abundance depending on geographical location (41). This bacterium resides within the mucus layer where it binds to complex type O-glycans (core 2–4) (42). In addition to binding to mucin glycans, *A. muciniphila* also enzymatically cleaves mucin glycans using glycosyl hydrolases (25, 32, 34, 37, 43–47). Transcriptomics show that 3% of the *A. muciniphila* genome is devoted to mucin degradation (48), suggesting that mucin degradation is pivotal in this bacterium’s lifestyle. *A. muciniphila* has GH33, GH16, GH29, GH95, GH20, GH2, GH35, GH84, and GH89, which allow *A. muciniphila* to cleave sialic acid, fucose, galactose, and N-acetylglucosamine (25). *A. muciniphila* possesses multiple glycosyl hydrolases, sulfatases, and a polysaccharide lyase that work cooperatively to break down mucin O-glycans (49–52). A recent study with a library of transposon mutants identified that many of these glycosyl hydrolases are redundant in *A. muciniphila* (43), suggesting that this bacterium has evolved a robust and flexible enzymatic toolkit to ensure efficient mucin degradation. Mucin degradation-related genes account for ~14% of the *A. muciniphila* genome (53). These glycosyl hydrolases are upregulated in the presence of mucus (54), and through the combined action of these enzymes, *A. muciniphila* can completely degrade mucin glycan structures down to the core N-acetylgalactosamine and hydrolyze up to 85% of mucin glycan structures (46, 49, 55). After cleavage, *A. muciniphila* consumes the mucin-derived monosaccharides N-acetyl-glucosamine and N-acetyl-galactosamine (48). *A. muciniphila* also possesses several proteases that degrade the mucin protein, also called mucinases (46, 56–61). Unlike glycosyl hydrolases that cleave sugar moieties from mucin O-glycans, mucinases target the peptide-rich regions of the mucin protein. Examples of *A. muciniphila’s* mucinases include Amuc_1434, AMUC_1438, Amuc_0627, and OgpA. The activity of these mucinases allows *A. muciniphila* to utilize mucin protein as a nitrogen source. After consuming mucin glycan and protein degradation products, these compounds accumulate in *A. muciniphila’s* internal compartments in a process that requires mucin utilization locus (MUL) genes, such as genes that encode pili and periplasmic protein complexes (43). *A. muciniphila* is considered to be a mucin specialist since the bacterium requires mucin-associated sugars for growth, and mucin glycosyl hydrolases are necessary for the bacterium to colonize the intestine *in vivo* when other microbes are present (43, 62–64). For the sake of simplicity in this review, we will assume that if other microbes are present in the intestine (i.e., if conventional animals are used as the model), *A. muciniphila* participates in mucin degradation.

## DIRECT EFFECT OF *A. MUCINIPHILA* ON GOBLET CELLS AND MUCUS PRODUCTION

Goblet cell responses to *A. muciniphila* and its metabolic byproducts vary depending on the context. Three studies using mouse intestinal organoids have noted that *A. muciniphila* products can upregulate goblet cell-produced MUC2. Kang et al. found that *A. muciniphila*-secreted protein Amuc_1409 increased MUC2 levels by quantitative PCR (qPCR) in apical out mouse intestinal organoids (65). He et al. found that propionate, which can be secreted by *A. muciniphila*, was able to increase MUC2 when added to the outside of apical side in mouse intestinal organoids (66). Additionally, Kim et al*.* found that the addition of cell-free supernatant from *A. muciniphila* to the outside of apical in mouse intestinal organoids increased MUC2 by qPCR (67). In contrast, Lukovac et al. found that application of cell-free supernatant from *A. muciniphila* to the outside of apical in mouse intestinal organoids significantly upregulated the transcription of genes involved in host metabolism, but surprisingly, no effects on goblet cell pathways were identified (68). Although mucin content was not examined, a study using pasteurized *A. muciniphila* on mucus-producing HT29-MTX-E12 cells identified an increased diversity of sialidated O-linked glycans in response to the bacterium (69), suggesting that *A. muciniphila* surface proteins may be able to impact mucin glycosylation. In mice with a complete gut microbiota, also known as conventional mice, administration of *A. muciniphila* has been shown to have both beneficial and detrimental effects on goblet cells and the mucus layer. These studies will be discussed later on in this review. Interestingly, when germ-free mice were mono-associated with *A. muciniphila*, *A. muciniphila* did not increase mucus-related genes (35) or change goblet cell number (70). Similarly, another study using IL-10 KO germ-free mice found that mono-association with *A. muciniphila* did not affect goblet cell numbers, MUC2 mRNA expression, or the mucus layer thickness (71). These studies suggest that *A. muciniphila* may be able to directly impact goblet cells by itself, but more research is required to fully tease out this interaction.

## *A. MUCINIPHILA* METABOLITES

*A. muciniphila* degrades both mucus glycan and mucin proteins and uses them as carbon and nitrogen sources to support growth. For example, *A. muciniphila* has been shown to ferment mucin-associated sugars, such as galactose, N-acetyl-glucosamine, and N-acetyl-galactosamine (48). *A. muciniphila* can also consume amino acids, such as threonine (72), which is a major amino acid in the mucin protein backbone. The main metabolites of mucus degradation by *A. muciniphila* are short-chain fatty acids (SCFAs). *A. muciniphila* can use mucin sugars to generate the SCFAs acetate and propionate (32, 48, 52, 73–78). Of the mucin-associated sugars, N-acetyl-glucosamine and N-acetyl-galactosamine are particularly potent stimulators of acetate and propionate (48). Acetate and propionate are important regulators of gut health, as they promote mucus production, stimulate hormones, suppress inflammation, regulate host metabolism, modulate histones, and protect the intestinal epithelium (66, 68, 79–83). *A. muciniphila* can also generate the branched chain fatty acids (BCFAs) iso-butyric acid and iso-valeric acid (76, 84), which can influence gut barrier function, immune response, and overall metabolic health. In addition to SCFAs and BCFAs, *A. muciniphila* secretes succinate (48, 73), 1,2-propanediol (48), diacyl phosphatidylethanolamine (84), glucagon-like peptide 1 (48), cell components Amuc_1100 (85), Amuc_2109 (86), Amuc_2172 (87), Amuc_1831/Protein 9 (P9) (88), and bioactive lipids (89). Similar to other Gram-negative bacteria, *A. muciniphila* is adept at generating extracellular or outer-membrane vesicles (90–97). It is unclear if these compounds, proteins, and vesicles are directly made from mucus, but since the culture conditions used to identify these compounds contain mucus, it is likely that many of these components are products of mucus degradation.

**Fig 2:**
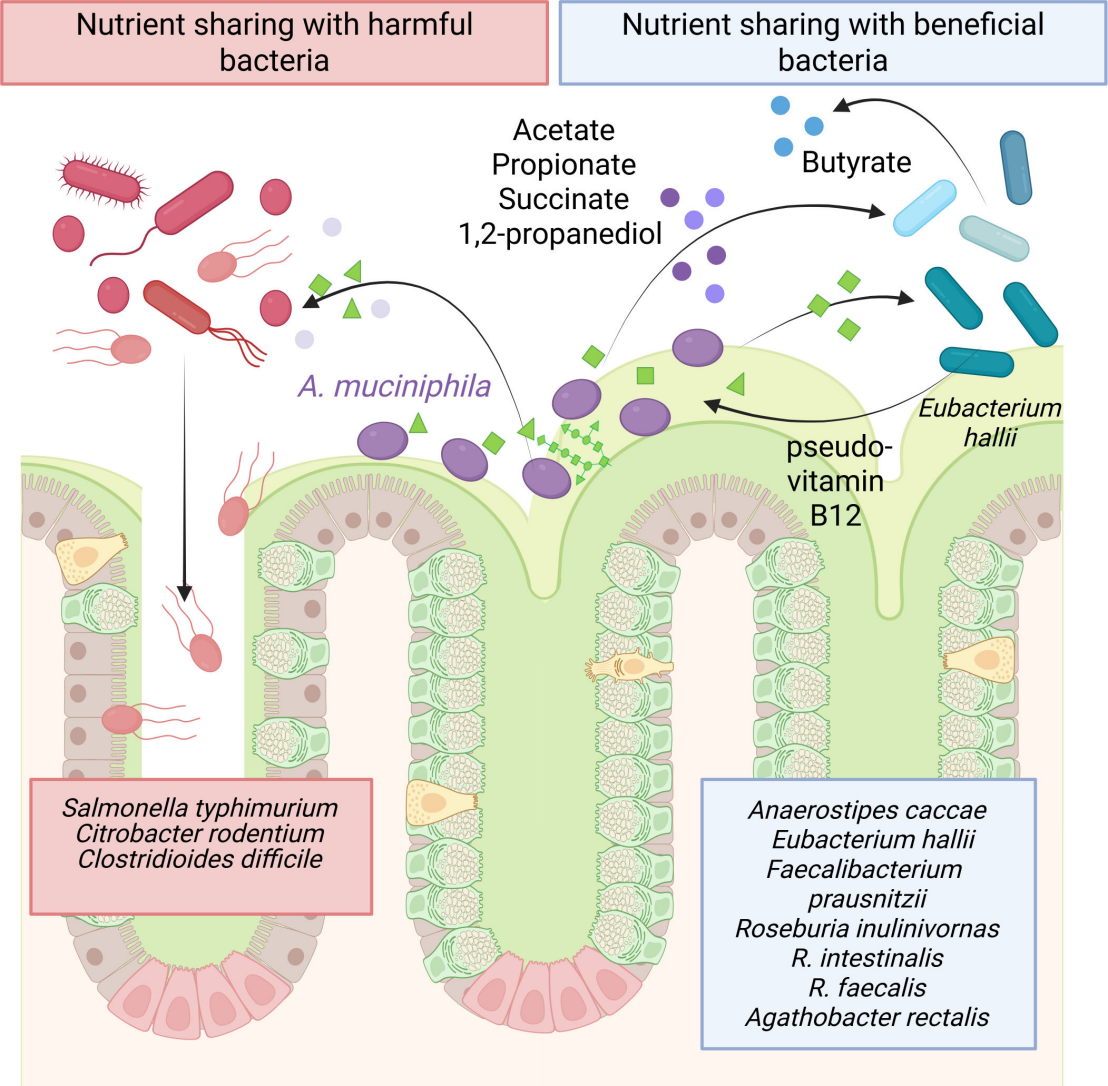
Diagram of *A. mucinphila’s* ability to share nutrient and participate in bacterial crosstalk with beneficial bacteria (*Anaerostipes caccae, Eubacterium hallii*, *Faecalibacterium prausnitzii, Roseburia inulinivorans, R. intestinalis, R. faecalis,* and *Agathobacter rectalis*) and harmful bacteria (*Salmonella typhimurium*, *Citrobacter rodentium*, and *Clostridioides difficile*).

Mucin degradation by *A. muciniphila* produces byproducts such as acetate, propionate, succinate, and 1,2-propanediol, which can be cross-fed to other bacteria within the gut (Fig. 2). This interaction fosters a complex ecosystem where metabolic byproducts from one species support the growth of another, enhancing microbial diversity. Microbiome analysis from dietary intervention studies suggests a co-occurrence of *A. muciniphila* with second-line butyrate producers, such as *Anaerostipes caccae, Eubacterium* species, *Faecalibacterium prausnitzii,* and *Roseburia* species (98–106). Several *in vitro* studies have confirmed this co-association and demonstrated that *A. muciniphila* can cross-feed commensal bacteria. Mucin degradation by *A. muciniphila* can cross-feed commensal microbes *Anaerostipes caccae, Eubacterium hallii*, and *Faecalibacterium prausnitzii* and promote the production of butyrate, an inducer of mucus synthesis and enterocyte/colonocyte growth (107–110). Co-culture of *A. caccae* and *A. muciniphila* increased the expression of mucin-glycan degradation genes in *A. muciniphila*, suggesting that certain gut microbes can elevate mucin-degrading enzymes (110). A recent paper by Shouker et al. demonstrated that *A. muciniphila’s* sialidases and fucosidases were specifically responsible for enabling nutrient sharing with other gut bacteria (44). This study found that sialic acid released from the mucin glycan did not contribute to *A. muciniphila* growth, but instead promoted butyrate production by co-cultured Clostridia, such as *Roseburia inulinivorans, R. intestinalis, R. faecis, Agathobacter rectalis*, and *Faecalibacterium prausnitzii* (44). In another bacterial interaction, *A. muciniphila*-generated 1,2-propanediol was used by *E. hallii,* and *E. hallii,* in turn, provided pseudovitamin B12 so *A. muciniphila* could generate propionate (109). As a result of these beneficial cross-feeding events, some groups have begun generating probiotic mixtures that contain *A. muciniphila* with other commensal microbes, like *A. hallii, Clostridium butyricum*, and *Bifidobacterium* species (111, 112). El Hage et al. demonstrated that the addition of propionate-producing bacteria *Lactobacillus plantarum, Bacteroides thetaiotaomicron, Ruminococcus obeum, Coprococcus catus, Bacteroides vulgatus, Veillonella parvula,* and *Akkermansia muciniphila* to clindamycin-depleted stool seeded human intestinal microbial ecosystems, which contained mucus, was able to increase the production of propionate, but not acetate or butyrate (113). These studies suggest that when commensal bacteria are present, *A. muciniphila* can cross-feed beneficial microbes and collectively benefit the gut. However, several studies also suggest that *A. muciniphila* can cross-feed pathobionts and pathogens and negatively impact the gut. In the upcoming sections, we will discuss how mucus degradation by this bacterium can contribute to both health and disease.

## DEGRADATION OF MUCINS BY *A. MUCINIPHILA* PROMOTES HOST HEALTH

**Fig 3:**
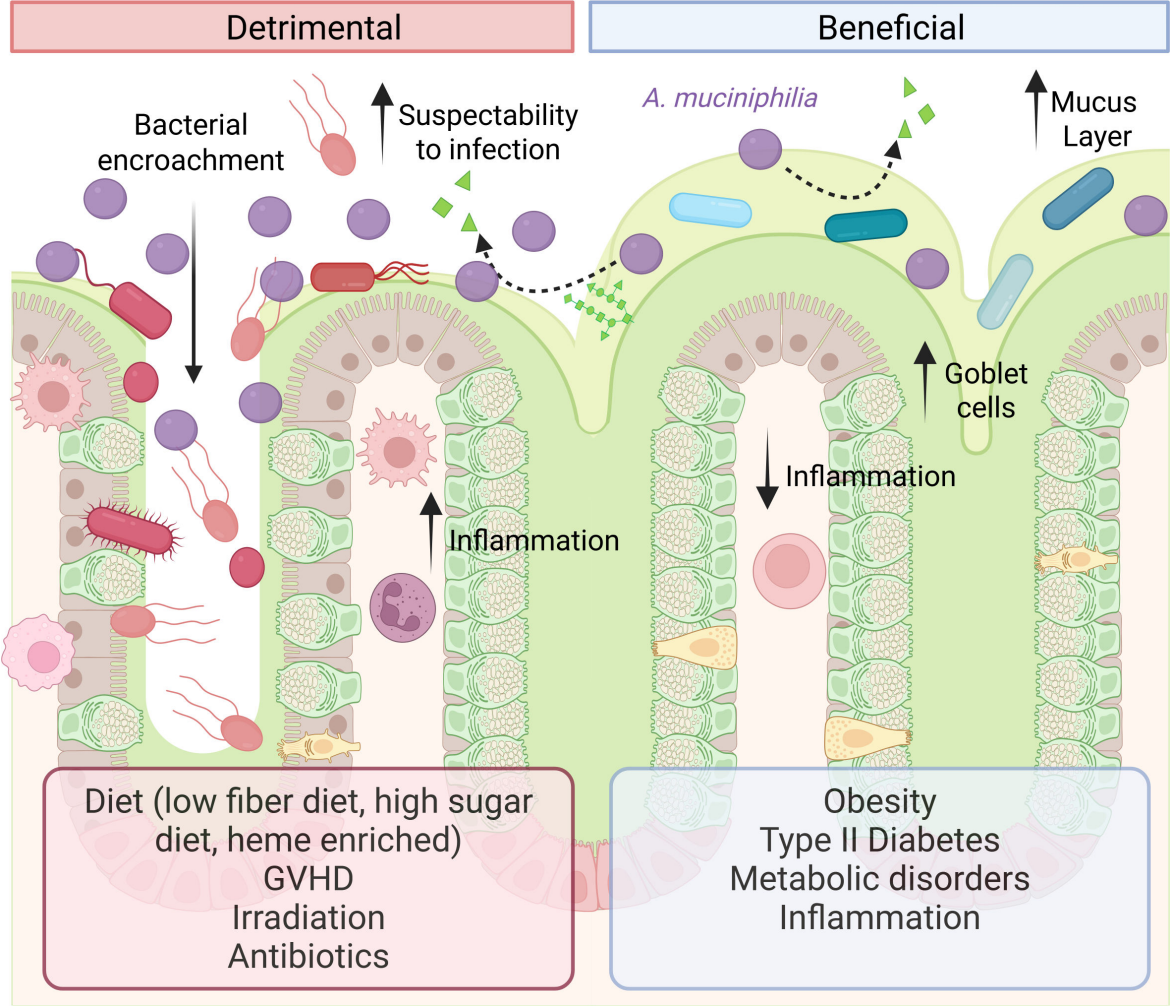
Diagram of *A. mucinphila’s* ability to affect host health through a variety of factors such as differentially changing the mucus layer, goblet cell number, inflammation, susceptibility to infection, and bacterial encroachment of the gut epithelium.

Multiple studies have demonstrated that *A. muciniphila,* in general, is associated with human health (Fig. 3). *A. muciniphila* levels are inversely correlated with disorders such as obesity, type 2 diabetes, inflammatory bowel disease (IBD), and intestinal inflammation in mice and humans (30, 54, 85, 97, 114–127). Obese individuals have been shown to have a significant reduction in *A. muciniphila* (114, 128–130), and *A. muciniphila* levels were inversely related to fasting glucose, waist-to-hip ratio, and subcutaneous adipocyte diameter (131). Subjects with higher *A. muciniphila* abundance exhibited the healthiest metabolic status in terms of fasting plasma glucose, plasma triglycerides, and body fat distribution (130, 131). Caloric restriction and dietary-exercise combined weight loss intervention of overweight individuals has been associated with elevated levels of *A. muciniphila* (129, 132), and individuals with higher baseline *A. muciniphila* displayed greater improvement in insulin sensitivity markers and other clinical parameters after calorie restriction (131). The Roux-en-Y gastric bypass weight-loss surgery has also been demonstrated to increase the relative number of *A. muciniphila* after 3 months of follow-up (133). The genus *Akkermansia* has further been shown to be enriched in athletes with a low body mass index (134, 135). Similarly, in mice, intermittent fasting elevates *A. muciniphila* and promotes gut health (136). These correlations have been confirmed in animal and human studies where oral administration of *A. muciniphila* reversed high-fat diet and obesity-induced metabolic disorders (54, 85, 116, 124, 137–142). For example, Everard et al. found that a high-fat diet reduced the inner colonic mucus layer of mice, but administration of *A. muciniphila* reduced this mucus thinning, lowered serum lipopolysaccharide, and reduced fat mass gain (116). These studies highlight the connection between *A. muciniphila* levels and weight.

In addition to obesity, *A. muciniphila* has been negatively correlated with type 2 diabetes in both humans and animal models, where diabetes patients and animals have a lower abundance of *A. muciniphila* (117, 143–150). A recent comprehensive high-level statistical analysis of multiple animal studies found that *A. muciniphila* administration significantly decreased weight gain by 10.4%, fasting blood glucose by 21.2%, significantly improved glucose tolerance by 22.1%, and increased blood insulin levels by 26.9% (151). In humans, a randomized, double-blinded, placebo-controlled study identified that supplementation with *A. muciniphila* to 95 participants with type 2 diabetes who were overweight or obese and drug-naïve had distinct effects in individuals based on the baseline levels of *A. muciniphila* (150). For example, individuals who had low baseline levels of *A. muciniphila* and received *A. muciniphila* treatment had reduced body weight, visceral, and total fat mass, and HbA1c compared with placebo controls. This study further confirmed this finding by transplanting human feces into germ-free mice (150). Additionally, metformin and dapagliflozin administration, which are used to treat type 2 diabetes, increase *Akkermansia* and improve glucose homeostasis (121, 152–156). New studies using glucagon-like peptide-1 (GLP-1) agonists like semaglutide (157, 158), liraglutide (159, 160), and tirzepatide (161) also demonstrate the ability to elevate *Akkermansia* abundance. Similarly, as reviewed in reference 162, in clinical trials with diabetic and obese individuals, supplementation of *A. muciniphila* has been shown to result in improved clinical and metabolic outcomes. Moreover, *A. muciniphila* has also been shown to improve glucose homeostasis and metabolic disease in mice fed a high-fat diet through the secretion of GLP-1 (163). As reviewed in reference 144, the improvement in glucose tolerance with *A. muciniphila* has been proposed to be related to reduced ER stress, which can modulate mucus production and the gut barrier (164).

Multiple studies have also shown that IBD patients have lower levels of fecal *A. muciniphila* (30, 126, 165–168). Png et al. identified that *A. muciniphila* levels were reduced by a mean of 92-fold and 172-fold in non-inflamed and inflamed regions of ulcerative colitis patients and reduced by 14.8-fold in inflamed regions of Crohn’s Disease patients (30). Two other papers identified significantly lower amounts of *A. muciniphila* in ulcerative colitis patients with active disease, but no significant difference between the healthy controls and the patients in remission, suggesting the possibility that reduction of *A. muciniphila* may play a role during active inflammation (167, 169). A study that specifically examined the microbiota of colonic mucus brushings found a decreased abundance of *A. muciniphila* in all four areas of the colon, cecum, transverse colon, left colon, and rectum from patients with active ulcerative colitis compared to healthy controls (170). This study further identified a positive association between *A. muciniphila* abundance and the percentage of sulfated mucin in the mucus gel layer (170), demonstrating a correlation between *A. muciniphila* levels and mucin degradation. Consistent with having a role in limiting inflammation in IBD, administration of *A. muciniphila* has been shown to beneficially regulate the immune system and suppress inflammation in mouse models (66, 125, 171–181). Although some of these effects are dependent on *A. muciniphila* strain, stand-alone administration versus supplemented administration, and host interaction, these studies still demonstrate a beneficial effect of *A. muciniphila* in colitis models. Other recent studies have identified *A. muciniphila* as being able to counteract the deleterious effects of dietary emulsifiers (182). The effects of *A. muciniphila* in the intestine extend to other organs in the gastrointestinal tract, as *A. muciniphila* administration ameliorates fatty liver disease and alcoholic liver disease and reduces liver injury (183–190) and improves beta-cell function in the pancreas (191).

*A. muciniphila* has been shown to have a direct effect on the gut epithelium. *A. muciniphila* has been shown to increase Lgr5+ intestinal stem cells and antimicrobial Paneth cells (67); thereby modifying the composition of the gut epithelium. Kim et al. linked *A. muciniphila* to the ability to promote intestinal stem cells and increase intestinal epithelial regeneration (67). This is consistent with other publications, which demonstrate that *A. muciniphila* drives intestinal wound repair (66, 192, 193). In the setting of conditions that reduce mucus-producing goblet cells, *A. muciniphila* and its byproducts have been shown to limit this reduction and retain goblet cells (67, 121, 125, 178, 194–196). For example, *A. muciniphila* was shown to reduce the effects of colonic mucus shrinkage and enhance immune activation due to aging in an accelerated aging mouse model, Ercc1^−/Δ7^ mice (194). Additionally, administration of prebiotics, which elevate *A. muciniphila* and other commensal microbes, has also been shown to elevate mucus production (98). These studies highlight the many host-associated benefits of *A. muciniphila*.

## DEGRADATION OF MUCINS BY *A. MUCINIPHILA* IS DETRIMENTAL TO HOST HEALTH

Although *A. muciniphila* and its metabolites positively influence metabolic disorders, several studies have found that mucus degradation by *A. muciniphila* also contributes to thinning the mucus layer. If mucus degradation is excessive or uncontrolled, it can erode the protective mucus layer and expose intestinal cells to harmful substances, including bacterial toxins, inflammatory compounds, or pathogenic microbes, increasing the risk of colitis and infection (29, 197). For example, in mice on a normal chow diet, *A. muciniphila* was shown to degrade and reduce the inner protective mucus layer, although not statistically significant, the data trend to display a thinning of the mucus layer due to *A. muciniphila* treatment (116). In mice treated with antibiotics and then administered oral *A. muciniphila* to create an “over-colonized” *A. muciniphila*, there were also significantly lower levels of mucus and increased inflammation (31). Several studies have demonstrated that in the setting of a low-fiber diet, *A. muciniphila* is elevated and associated with a decreased mucus layer and negative outcomes (29, 198, 199). For example, Desai et al. demonstrated that a low fiber diet expanded *A. muciniphila* levels, thinned the inner mucus layer, and promoted heightened susceptibility to the pathogen *Citrobacter rodentium*, which resembles infection by enteropathogenic *Escherichia coli* (EPEC) and enterohemorrhagic *E. coli* (EHEC) in humans (29). Wolter et al. (199) went on to use a functionally characterized, 14-member synthetic human microbiota community in gnotobiotic mice to deduce which bacteria and functions were responsible for the pathogen susceptibility. By removing specific strains of bacteria from microbial communities, focusing on mucolytic bacteria, this study found that *A. muciniphila* specifically enhanced host susceptibility to *C. rodentium* infection during fiber deprivation, as exclusion of *A. muciniphila* from the synthetic community was sufficient to prevent severe infection (199). The enhanced pathogen susceptibility was not due to an altered host immune system or pathogen responses, but it was driven by a combination of increased mucus penetrability and altered activities of *A. muciniphila* and other community members. When the mice were returned to a fiber-rich diet, the presence of *A. muciniphila* reduced the pathogen load (199), highlighting the context-dependent effects of this mucin specialist. In a similar vein, Parrish et al. used the same 14-member synthetic human gut microbiota and found that mice on a fiber-free diet with elevated *A. muciniphila* had enhanced susceptibility to food allergic responses and enhanced colonic inflammation (198). Using the same dropout experiments, this study found that *A. muciniphila* was specifically responsible for this sensitivity. These studies demonstrate that in fiber-deplete diets, *A. muciniphila* can have detrimental effects on the host.

In addition to fiber, glucose has been shown to influence the levels of *Akkermansia in vivo* (197). A recent study by Khan et al. found that mice fed 10% glucose or fructose in drinking water had elevated Akkermansiaceae and specifically *A. muciniphila* compared to vehicle control-treated animals. Analysis of the colonic mucus layer revealed that the inner mucin layer of glucose-treated mice was thinner, and the gut bacteria were in closer proximity to the epithelial layer compared to the control animals (197). In addition to having a depleted mucus layer, these sugar-treated mice also developed severe colitis when they received dextran sulfate sodium (DSS). This phenotype was microbiome dependent, as the phenotype could be transferred by fecal microbiota transplant (FMT) to germ-free recipient mice and IL-10 KO mice, and antibiotic administration ablated the phenotype (197). Similar to the glucose diet, mice fed high fructose diets have also been found to have an expansion of *Akkermansia* (195, 200). When examining the effects of diets with high or low calcium phosphate levels in rats, Fuhren et al. identified that low dietary calcium phosphate levels also promoted the abundance of *Akkermansia* (201). Another study found that mice given a heme-enriched diet had an eightfold increase in *A. muciniphila* in their feces compared to control mice on a regular diet (202). Mice on the heme-enriched diet also had increased levels of sulfides, particularly trisulfides, and had mucus with open polymeric MUC2 networks that could allow increased bacterial degradation (202). The authors speculated that these trisulfides could serve as a novel marker of colonic mucus degradation and a proxy for a reduction in the mucus barrier. These data suggest that certain diets can elevate *Akkermansia*, decrease the mucus layer, and have negative impacts on the host.

In addition to diet, several studies have examined the impact of *A. muciniphila* in murine transplant models and graft versus host disease (GVHD) in mice and humans (203–206). In these studies, broad-spectrum antibiotics ampicillin, imipenem-cilastatin, and meropenem altered the gut microbiota and elevated *A. muciniphila*. Corresponding with increased *A. muciniphila*, mice with GVHD exhibited a depletion of the colonic mucus layer, increased epithelial damage, and elevated GVHD-related mortality rate (204). Changes in bacterial compositions revealed that broad-spectrum antibiotics suppressed the anaerobic commensals such as *Lactobacillus, Clostridium,* and *Blautia* and increased the abundance of pathobionts like *Enterococcus*. Another study found that *A. muciniphila* was further elevated in a murine GVDH model on a high stearic acid diet, which was associated with worse GVDH-associated mortality (206). Administration of antibiotics designed to target *A. muciniphila* and other pathobionts was found to reduce *A. muciniphila* levels and attenuate GVHD (206). This study also found that GVHD patients had significantly higher concentrations of *A. muciniphila* compared with non-GVHD patients. In addition to GVDH, *A. muciniphila* is one of the major groups elevated in irradiated mice, and microbial species transplanted from irradiated mice to new murine hosts also result in severe intestinal damage compared to those that received naive microbes (207). Analysis of the microbiome of these mice revealed a reduction in commensal Clostridia and an upregulation of pathobiontic Turicibacter and Proteobacteria (207). Another study examining irradiation and melphalan-induced neutropenia identified elevated *A. muciniphila* levels and decreased commensal Bacilli and Erysipelotrichales in patients who developed fever (208). Additionally, this same study found that caloric restriction of mice also expanded *A. muciniphila* and thinned the colonic mucus layer. Antibiotic treatment to eradicate *A. muciniphila* before caloric restriction preserved colonic mucus, while *A. muciniphila* reintroduction restored mucus thinning (208). This paper went on to demonstrate that irradiation of mice also increased *Akkermansia* (208). Evidence of a preserved mucus layer, suppressed translocation of flagellin, reduced inflammatory cytokines in the colon, and improved thermoregulation was all shown from irradiated mice treated with an antibiotic targeting *A. muciniphila* (208). These data align with cell line models, which demonstrate that in the absence of a mucus layer, *A. muciniphila* stimulates the epithelial pro-inflammatory cytokine IL-8 (209). These studies suggest that *A. muciniphila* may participate in a deleterious crosstalk with other bacteria in the setting of GVHD and irradiation.

*Akkermansia* and in many cases specifically *A. muciniphila* is elevated in several conditions with negative associations, such as antibiotic use (202, 210, 211), proton-pump inhibitor and aspirin use (212), radiation-associated inflammation (207, 213–215), pharmaceutical excipient PEG400 administration (216), human multiple sclerosis (217–227), human Parkinson’s disease (228–233), human Alzheimer’s disease (234–236), human epilepsy (237), human immunoglobulin A nephropathy (238), human bladder cancer (239), carriers of extended-spectrum β-lactamase-producing Enterobacteriaceae (240), murine Fragile X syndrome (241), and murine models of spontaneous colitis (242, 243). Currently, it is unclear why *Akkermansia* is elevated in these conditions. Despite the fact that *Akkermansia* is elevated in some diseases and disease models, it should be noted that the presence of *Akkermansia* is not always deleterious. For example, some mouse and zebra models have found that administration of *A. muciniphila* can improve outcomes of Alzheimer’s disease in these models (244–246). However, in some cases, the administration of *A. muciniphila* exacerbates disease (31, 243, 247, 248). For example, studies have found that supplementation of *A. muciniphila* worsened the colitis in IL-10 KO mice (243) and exacerbated necrotic enteritis in chickens (247). In a model of small intestine injury caused by a proton pump inhibitor and aspirin, *B. bifidum* was found to limit mucus degradation by *A. muciniphila* and reduce the intestinal damage (212). Other studies report elevated *A. muciniphila* during colitis (249–251), and the presence of *Akkermansia* was also found to correlate with FMT treatment failure in a mouse model of colitis (252). These studies highlight that the presence of *Akkermansia* is not always beneficial.

## CONFLICTING FINDINGS ON *A. MUCINIPHILA* IN INFECTIOUS DISEASE

Interestingly, *A. muciniphila’s* impact on infectious agents, such as *Salmonella, Citrobacter rodentium,* and *Clostridioides difficile*, has been identified to have both beneficial and detrimental effects. In terms of beneficial aspects, oral delivery of *A. muciniphila* in young chicks improved *Salmonella pullorum* infection by limiting chick weight loss, decreasing mucosal damage, and increasing epithelial cell proliferation (253). In a similar vein, *A. muciniphila* metabolites from cultures grown with mucus inhibited the growth and biofilm capacity of *Salmonella enterica serovar* Typhimurium *in vitro* (254), and oral delivery of *A. muciniphila* to conventional mice reduced colonization of *S*. Typhimurium and limited intestinal damage *in vivo* (255). In terms of detrimental effects, using a simplified gut microbiota (SIHUMI) in gnotobiotic mice, one study found that *A. muciniphila* was specifically responsible for exacerbating *S. Typhimurium* infection in mice (256). Another paper observed that infection with *S*. Typhi upregulated *Akkermansia* species (257). These studies suggest mixed findings on the role of *Akkermansia* in *Salmonella* infection.

Similar to the findings with *A. muciniphila* and *Salmonella*, there are also conflicting findings with *A. muciniphila* and *Citrobacter* and *C. difficile. Akkermansia* has been shown to be elevated during the peak of *C. rodentium* infection in mice (258). *A. muciniphila* speficially was expanded in the colonic mucus layer of ILC3-deficient mice, and *A. muciniphila* and its metabolite succinate enhanced virulence factors in *C. rodentium*, specifically *tir* and *ler,* and increased the susceptibility of mice to *C. rodentium* infection (259). As described in the determinantal effects section, in gnotobiotic animals with defined communities and conventional mice on a low fiber diet, *A. muciniphila* worsened *C. rodentium* infection (29, 199). However, when the mice were returned to a high fiber diet, *A. muciniphila* reduced the pathogen load (199). Another study found that hyaluronan elevated *Akkermansia* and reduced *C. rodentium* in conventional mice (260). This same study found that administration of *A. muciniphila* limited animal weight loss, reduced inflammation, and improved the epithelial architecture during *C. rodentium* infection (260). *C. rodentium* is closely related to EPEC and EHEC and is often used to model these organisms in animals (261). Interestingly, in conventional mice and piglets infected with enterotoxigenic *E. coli* (ETEC), a transplant of stool containing *A. muciniphila* was able to reduce intestinal injury in these animals (262). *In vitro* in apical-out intestinal organoids, *A. muciniphila* was able to activate the Wnt/β-catenin signaling pathway (262), suggesting a possible mechanism by which *A. muciniphila* was able to reduce ETEC-induced damage.

The findings with *C. difficile* mirror some of the same conclusions as *C. rodentium. C. difficile* consumes many mucin-associated glycans, particularly sialic acid and N-acetyl-glucosamine (263, 264). Administration of a cocktail of mucin-consuming bacteria, *Ruthenibacterium lactatiformans, Alistipes timonensis, Muribaculum intestinale, Bacteroides* sp., and *A. muciniphila,* reduced *C. difficile* growth *in vitro* and *in vivo* (264). *In vivo,* this mucin-consuming cocktail also limited toxin-mediated intestinal inflammation during *C. difficile* infection (264), suggesting that mucin glycan consumption could be a method to outcompete *C. difficile*. Similarly, another study found that mucin-consuming *Bifidobacterium breve, Bacteroides ovatus,* and *A. muciniphila* all worked together to reduce *C. difficile*-induced cell damage in a murine model (265). In a separate study, administration of *A. muciniphila* by oral gavage to conventional mice reduced the weight loss, diarrhea, and inflammation observed in *C. difficile*-infected mice (266). This effect was associated with increased short-chain fatty acids and an improvement in bile acid profiles. In a gnotobiotic model with a defined 21-member microbial community that is unable to resist *C. difficile*, infection with *C. difficile* was found to decrease *A. muciniphila* levels (267). However, conversely, *A. muciniphila* has been shown to be elevated in patients with *C. difficile* infection (268–270). Additionally, another paper found in *C. difficile*-infected mice showed that *Akkermansia* was elevated in mice that were IgG negative compared to IgG positive (271). *In vitro*, mucin degradation by *A. muciniphila* was shown to cross-feed *C. difficile* and regulate its flagella expression (263). These findings suggest that *A. muciniphila* may participate in both suppressing and elevating enteric pathogens.

In addition to studies examining *A. muciniphila* in bacterial infections, *A. muciniphila* has been studied in the setting of parasitic worm, or helminth, infection, as reviewed in reference 272. According to a recent meta-analysis, *Akkermansia* levels are elevated in people with *Enterobius* infection compared to individuals without infection (273). Another study in Sri Lanka identified that individuals infected with GI helminths had an increase in *A. muciniphila* compared to uninfected individuals (274). In mouse models, two studies have shown that infection with *Heligmosomoides polygyrus* increased the relative abundance of *A. muciniphila* (275, 276). The elevated *A. muciniphila* was particularly pronounced in mice receiving a high-fat diet and *H. polygyrus* (275). Moreover, mice that received an FMT from helminth-infected donors had increased susceptibility to *C. rodentium* infection (276), suggesting that the gut microbiota, including *Akkermansia*, can participate in exacerbated infection. The mouse helminth, *Trichuris muris*, also increased the abundance of the phylum Verrucomicrobiales (272), and the generalist *Trichinella spiralis* was also found to increase the abundance of *Akkermansia* (274, 277, 278). In helminth infections, the body’s response often includes an increase in mucus production and fluid secretion to help expel the parasites, as reviewed in reference 279. The elevated abundance of *A. muciniphila* may be a result of an increase in intestinal mucus, which serves as a nutrient source for *A. muciniphila*. In the setting of helminth infection, *A. muciniphila* has been shown to subsequently decrease adult worm burden and benefit the host (272). One study in *T. spiralis* infection found that administration of β-Glucans further increased *Akkermansia* levels and led to an overall increase in mucus-positive goblet cells in the small intestine (280). This same study went on to orally administer *A. muciniphila,* and they also observed a reduction in *T. spiralis* and increased mucus-filled goblet cells. It is unclear how mucin degradation plays a role in *A. muciniphila’s* effects on helminth infection, but this is an interesting area for future research.

**Fig 4:**
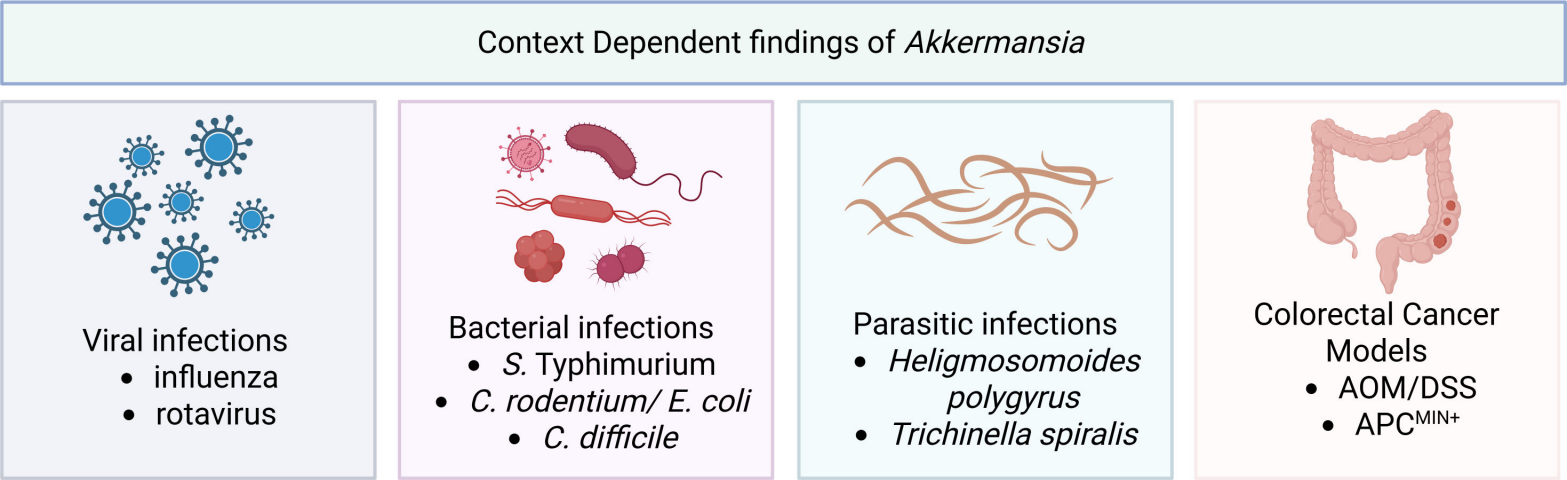
Diagram of *A. mucinphila’s* context-dependent effects on host health, such as its ability to change disease outcomes associated with viral infections, bacterial infections, parasitic infections, and colorectal cancer.

Viral infections have also been noted to cause alterations in *Akkermansia* levels. In neonatal mouse pups, rotavirus infection elevated *Akkermansia* abundance at the beginning of infection, and this finding was associated with decreased mucus-filled goblet cells (281). Rotaviruses attach to intestinal enterocytes via specific glycans, as covered in the following reviews (282, 283). Several viruses, including rotavirus, bind to sialic acid and galactose residues that are also found on mucin glycans, and the VP8* domain of rotavirus has been shown to bind to mucin glycans containing these residues *in silico* (281). Rotavirus replication has been shown to be inhibited by the addition of intestinal mucins (281, 284). However, mucus that was degraded by *A. muciniphila* was shown to have reduced potential to inhibit rotavirus (281), suggesting that mucin glycans could serve as a decoy for rotavirus cell entry. *Akkermansia* levels are also elevated in H7N9 influenza infection in mice (285) and COVID-19 patients (286). Oral administration of live *A. muciniphila* to mice with H7N9 influenza virus did not affect survival rates or weight loss, but it did improve lung histology (285). There are some reports that *Akkermansia* is elevated in coronavirus infection, such as severe acute respiratory syndrome coronavirus 2 (287). Interestingly, coronaviruses are known to bind to sialic acid, and Yang et al. demonstrated that coronaviruses can bind to MUC2 from porcine intestinal organoids (288). The addition of the organoid mucus to coronaviruses, transmissible gastroenteritis coronavirus (TGEV) and porcine epidemic diarrhea virus (PED) reduced their infectivity in this study (288). In this setting, the removal of sialic acid glycans by *Akkermansia* could potentially enhance the infectivity of coronavirus. These studies highlight the significant variability in studies involving *Akkermansia* and infectious disease (Fig. 4).

## CONFLICTING FINDINGS ON *A. MUCINIPHILA* IN COLORECTAL CANCER

Several studies have also reported conflicting results about the effects of *Akkermansia* and *A. muciniphila* in colorectal cancer. In terms of beneficial aspects, one study by Wang et al. revealed that *A. muciniphila* abundance was significantly decreased by 16S rDNA sequencing in patients with colorectal cancer (289). This same study found that administering pasteurized *A. muciniphila* or recombinant surface protein Amuc_1100 by oral gavage to azoxymetane and dextran sodium sulfate (AOM/DSS) colitis-associated colorectal cancer mice resulted in decreased tumorigenesis. In the same study, oral administration of either pasteurized *A. muciniphila* or its recombinant surface protein Amuc_1100 significantly reduced tumorigenesis in mice with AOM/DSS-induced colitis-associated colorectal cancer (289). In another AOM/DSS colitis-associated colorectal cancer model, *A. muciniphila* was found to significantly decrease as tumorigenesis progressed, and oral delivery of *A. muciniphila* secreted extracellular vesicles was able to blunt the progression of tumorigenesis, perhaps through the recombinant protein Amuc_2172 (87). They further validated the anti-cancer effects of Amuc_2172 in the spontaneous tumorigenesis Apc^min/+^ mouse model and a subcutaneous injection of murine colon cancer CT26 cells in BALB/c mice (87). In addition, Fan et al*.* found a significant reduction in *A. muciniphila* abundance in colorectal cancer patients from two independent clinical studies and the GMrepo database (290). The authors also used a spontaneous tumorigenesis Apc^min/+^ mouse model and found that supplementation of viable *A. muciniphila* orally was also able to significantly suppress tumorigenesis. This study went on to show that supplementation of viable *A. muciniphila* orally also reduced the growth of implanted HCT116 or CT26 tumors in BALB/c nude mice (290).

Other beneficial effects of *A. muciniphila* can be seen in colorectal cancer models with therapies such as PD-1 or FOLFOX. Wang et al*.* used a model of subcutaneous injection of CT26 cells in BALB/c mice and found that oral administration of outer membrane vesicles derived from *A. muciniphila* was able to increase the efficacy of PD-1-based immunotherapy in colorectal cancer (196). Hou et al*.* also found by 16S rDNA sequencing that there was a significant increase in the abundance of *A. muciniphila* in individuals treated with FOLFOX, which was positively associated with the therapeutic outcome (291). The authors also utilized a subcutaneous injection of CT26 cells in the BALB/c mouse model, in which they described a significant increase in FOLFOX inhibition effects on tumor volume when combined with increased *A. muciniphila* colonization (291). These studies demonstrate that *A. muciniphila* can reduce colorectal cancer tumor burden and potentially synergize with cancer therapies.

On the other side, there have been reports of *A. muciniphila* and *Akkermansia* enhancing inflammation and colon cancer development. In an AOM/DSS model of cancer, tumor-bearing mice were found to harbor higher levels of *Akkermansia* (292). Two studies using C57BL/6 mice with AOM/DSS found that supplementation of *A. muciniphila* following antibiotics disrupted the microbial community, elevated inflammatory responses, and promoted colitis-associated colorectal cancer in mice (248, 293). Both studies suggested that *A. muciniphila* treatments resulted in decreased mucus levels (248, 293). Another study using an ectopic CT26 tumor model of colon cancer identified that tumor-bearing mice had elevated levels of *Akkermansia* (31). Using FabplCre; Apc^15lox/+^ mice, Dingemanse et al*.* demonstrated that oral gavage of *A. muciniphila* elevated the total number of tumors (294). Interestingly, in this animal model, the presence of *A. muciniphila* was associated with an increase in mucus-positive goblet cells and an increase in the inner mucus layer (294). Moreover, Baxter et al. reported that co-colonization of *Akkermansia* and *Bacteroides* in germ-free C57BL/6 mice treated with FMTs from human samples and subsequently exposed to AOM/DSS resulted in increased tumor formation (295). In humans, a study by Sanapareddy et al. found that the relative abundance of the phylum Verrucomicrobia was significantly increased in mucosal biopsies of patients with colorectal adenomas (296). Consistent with these results, Weir et al*.* uncovered that *A. muciniphila* is four times more frequent in colorectal cancer patients versus healthy adults (297). These studies point to *A. muciniphila* being elevated in colon cancer and exacerbating tumorigenesis. Clearly, the role of *A. muciniphila* in colorectal cancer remains complex and context-dependent.

## VARIABLES THAT CAN IMPACT *A. MUCINIPHILA* HOST INTERACTIONS

The diverse experimental models used to study *A. muciniphila* can make it challenging to interpret the effects of *A. muciniphila* on the host, as outcomes can vary significantly depending on factors like host genetics, diet, microbiota composition, and medication use. For example, while many studies have used C57BL/6 mice, a number of investigations have also employed other mouse strains, including Swiss Webster, BALB/c, DBA/2J, FVB/NJ, and sv129. The host genetic background plays a large role in regulating the gut microbiota composition. Ahn et al. identified that BALB/c, DBA/2J, and FVB/NJ mice had unique bacterial responses to a high fructose diet and that the gut microbiome of C57BL/6 mice and FVB/NJ mice had much higher levels of *Akkermansia* following a fructose diet compared to DBA2/J mice (200). In addition to mouse background, many studies use KO mice, including TLR2, PINK1, fmr1, Nlrp6, IL-18, IL-18R, IL-10, IL-1β, IL-1R, Fut8+, Apoe, Kcna1 KO mice, etc. as well as rat, chickens, and pig models (71, 241, 243, 245, 257, 298–308). The genetics and microbiomes of these models can vary significantly, and it is possible that conflicting data could arise from different models. Additionally, factors such as animal facility, co-housing, and cage bedding can have a significant impact on the gut microbiota (309–317). For example, Choo et al. reported that when C57BL/6 mice were transferred from a commercial supplier to a research facility, the abundance of *Akkermansia* markedly declined in the G1 offspring and remained low for at least six subsequent generations (309).

The baseline microbiome of the host can also impact mucus composition, which in turn may indirectly affect the abundance of *A. muciniphila*. Jakobsson et al. (316) identified two distinct specific pathogen-free (SPF) C57BL/6 mouse colonies housed within the same vivarium that exhibited differences in gut microbiota composition and mucus structure and function. Notably, the inner mucus layer in one colony was more permeable to fluorescent beads the size of bacteria (316), suggesting that microbial colonization patterns can modulate the architecture and barrier properties of the colonic mucus layer. These findings underscore how subtle variations in host-microbe interactions can reshape the biophysical properties of the mucus barrier and establish distinct ecological niches that may support or limit the growth of mucus-associated bacteria like *A. muciniphila*.

In addition to genetic, environmental, and microbiome influences, diet plays a large role in shaping the gut microbiome and controlling the levels of *Akkermansia*. Multiple dietary compounds and diets have been shown to increase the abundance of *Akkermansia*, including fructo-oligosaccharides (116, 138, 318, 319), arabinoxylan (98), stachyose (320), betaine (321), polyamines (322), green tea powder (323, 324), caffeic acid (325), conjugated linoleic acid (326), polyphenols (100, 327–333), polymethoxyflavones (334), puerarin (335, 336), oat bran (337, 338), whole-grain barley (339), red pitaya betacyanins (340), ferric oligosaccharides and polysaccharides (341, 342), Açai (343), rhubarb extract (344), pomegranate extract (345), berberine (346), capsaicin (346–350), resveratrol (351–353), epigallocatechin-3-gallate (354) hydroxysafflor yellow A (355), ketogenic diet (301, 356), low fiber diet (29, 198, 199, 357), FODMAP diet (104, 358), among other compounds (346, 359). Additionally, several dietary compounds and diets can decrease *Akkermansia* levels, including non-caloric artificial sweeteners (360), dietary emulsifiers (182), flaxseed (361), Yukihikari rice powder (362), high-fat diets (116, 121, 363, 364), and a westernized diet (365). Apart from diet, medications are known to impact the levels of *Akkermansia*. As previously noted, many antibiotics (210, 366–368) and drugs like metformin (121, 153, 155, 369) and melphalan (209) increase *Akkermansia* levels, while compounds like the proton pump inhibitor omeprazole can increase or decrease *Akkermansia* (212, 370). It is clear that multiple factors can influence the levels of *Akkermansia,* and conflicting findings about the effects of *A. muciniphila* could be related to the animal genetics, facility, diet, or medications.

## CONCLUSION

These findings underscore the dual nature of *Akkermansia muciniphila* and its role in mucin degradation. Under homeostatic conditions, *A. muciniphila* breaks down mucins to produce beneficial metabolites, such as the short-chain fatty acids acetate and propionate, the branched-chain fatty acids iso-butyric and iso-valeric acid, as well as succinate and 1,2-propanediol. These metabolites can stimulate host responses and support cross-feeding by other commensals to enhance butyrate production and promote gut health. While *A. muciniphila* is often associated with increased goblet cell numbers and thicker mucus layers in mice with a conventional microbiota, studies in mono-associated mice reveal that *A. muciniphila* alone does not increase goblet cells, suggesting that interactions with other microbes are essential to fully realize its mucin-promoting effects.

Conversely, under conditions such as low-fiber, high-sugar diets or in the context of a disrupted microbial community, *A. muciniphila* may contribute to mucus thinning and the release of mucin-derived oligosaccharides that can fuel the growth of pathobionts or pathogens, potentially exacerbating inflammation and infection. Based on the current body of evidence, we propose that *A. muciniphila* may be most beneficial when paired with a fiber-rich, low-sugar diet and a diverse microbial community. This review highlights the need to better understand mucin degradation and microbial community dynamics in order to guide future therapeutic applications of *A. muciniphila*.
